# Prevalence and Pattern of Soil-Transmitted Helminthic Infection among Primary School Children in a Rural Community in Imo State, Nigeria

**DOI:** 10.1155/2015/349439

**Published:** 2015-09-07

**Authors:** Kelechi Kenneth Odinaka, Emeka Charles Nwolisa, Francis Mbanefo, Alfreda Chinekwu Iheakaram, Seline Okolo

**Affiliations:** ^1^Department of Paediatrics, Imo State University Teaching Hospital, P.O. Box 1644, Orlu, Imo State, Nigeria; ^2^Department of Paediatrics, Federal Medical Centre, Owerri, Imo State, Nigeria; ^3^Department of Paediatrics, University of Jos Teaching Hospital, Plateau State, Nigeria

## Abstract

*Background.* Soil-transmitted helminthic infection is a common public health challenge of primary school children in resource challenged and developing countries. Our aim was to determine the prevalence and pattern of soil-transmitted helminthic infection among primary school children in a rural community in Imo State, Nigeria. *Method.* The study involved a cross-sectional survey of 284 primary school children in a rural community. *Results.* The overall prevalence of soil-transmitted helminthic infection (STHI) was 30.3%. Of all STHIs, hookworm was the commonest geohelminth observed, 81 (94.2%). The prevalence of soil-transmitted helminthic infection was more in males, 58 (38.4%), than in females, 28 (21.1%). This difference was statistically significant (*P* = 0.001). *Conclusion.* The high prevalence rate of soil-transmitted helminthic infection amongst the study population is worrisome. There is need for organized and routine deworming among school children in the community.

## 1. Introduction

Soil-transmitted helminths thrive and persist in human communities in which poverty, inadequate sanitation, lack of access to health care, and overcrowding are entrenched [1]. Additionally, the habits of bare feet on sand and eating unwashed fruits and vegetables also encourage the transmission of helminthic infection [2]. These habits occur in most rural communities and urban slums in resource challenged and developing countries including Nigeria. The World Health Organization (WHO) estimates that over two billion people are infected with one or more soil-transmitted helminths, mainly* Ascaris lumbricoides*, hookworm, and* Trichuris trichiura* [3]. School-aged children have been shown to be the population at greatest risk of acquiring infections with roundworm, hookworm, and whipworm infections [1, 4]. The preponderance of helminthic infection in school-aged children makes this subgroup a good target for helminth control programmes in the general population and schools provide good opportunities for implementation of control programmes [5, 6]. Intestinal helminthiasis is often associated with reduced physical activity and may worsen the already compromised nutritional status of the school-aged children in rural communities.

The prevalence of soil-transmitted helminthiasis differs from region to region. Although several studies [7, 8] have been conducted on the prevalence of intestinal helminthiasis in Nigeria, there are still localities including the study area for which epidemiological information is not available. WHO recommends that a baseline survey in school children be done to determine prevalence of worm infestation before instituting any worm control programme and treatment should be given according to the survey [9]. Presently, there is no national school-based helminth control programme in Nigeria. In the past, there have been sporadic and uncoordinated deworming programmes instituted by politicians and philanthropists. This study sought to determine the prevalence and pattern of soil-transmitted helminthiasis. This study will serve as a baseline for any future evaluation of proposed regular deworming programmes at schools in the area and the state in general.

## 2. Method

### 2.1. Study Area

The study was conducted in Amaruru, Orsu Local Government Area (LGA) of Imo State in Southeastern Nigeria. The community lies within the tropical rain forest zone of Africa and has the characteristic wet and dry seasons. Amaruru is predominantly an agrarian community with a mixture of traders, artisans, and civil servants. There are three government approved primary schools in Amaruru. None of the schools had water supply or toilet facility.

### 2.2. Study Design

The study was a cross-sectional, school-based, descriptive study. The participants for the study were selected using a systematic sampling technique. The study was performed between September and November 2012.

### 2.3. Study Population

The study population comprised children from primary one through primary six in all the three schools. The age of the children was determined from the school record.

### 2.4. Ethical Approval

Approval to carry out the study was obtained from the Ethics Committee of the Federal Medical Centre, Owerri, and Imo State Universal Basic Education Board. Written informed consent was obtained from parents/guardians of the subjects through the Parents-Teachers Association. Assent was also obtained from the children.

### 2.5. Laboratory Methods

Stool samples were examined using Stoll's dilution technique [10]. A small sized, clean, dry, and leakproof plastic container with a wide mouth and prelabelled with the subject's name and an identification number was issued to each recruited child. Students with the help of parents or guardians were instructed to bring to school stool specimens collected that morning. The stool sample was taken to the Federal Medical Centre, Owerri, parasitology laboratory within six hours after the stool had been passed, to be analysed for the presence of ova of soil-transmitted helminths. The pupils submitting stool samples were given pencils, erasers, or pens as incentives. All infected children were given a single dose of mebendazole (500 mg) at the expense of the researcher. Social classification of the children was based on the criteria set by Oyedeji (1985) [11].

### 2.6. Data Management and Analysis

Statistical analysis of the data was performed using the Statistical Package for the Social Sciences (SPSS) for Windows (version 16.0). Comparative analysis involving two categorical variables was done using chi-square test. The level of significance of each test was set at *P* < 0.05.

## 3. Results

A total of 288 children were recruited for the study; of these, 284 (98.6%) submitted stool samples while 4 (1.4%) did not submit stool sample. Data from these 284 subjects were analysed. The distribution of the subjects based on age group, gender, and socioeconomic class is shown in Table 1.

Of the 284 pupils studied, 86 (30.3%) had helminth ova detected in stool. The male subjects, 58 (38.4%), were more infected than the female subjects, 28 (21.1%). This difference was statistically significant (*P* = 0.001). Pupils who never washed their hands after defecation had the highest prevalence of helminthiasis (33.3%), followed by those who occasionally washed their hands (31.1%), while pupils who always washed their hands had the lowest prevalence (27.1%). The difference among the three groups in terms of personal hygiene was not statistically significant (*P* = 0.694). The data are shown in Table 2.

The prevalence of soil-transmitted helminthiasis was highest among subjects who sourced drinking water from the stream (43%), followed by those sourcing their drinking water from boreholes (38.4%). Subjects who sourced drinking water from springs had the lowest prevalence rate (1.2%). Among those infected, hookworm infection had a prevalence of (94.2%), followed by* Ascaris lumbricoides* (2.3%).* Trichuris trichiura* was not identified in the stool of study subjects. Infections with hookworm and* Ascaris lumbricoides* occurred in 3.5% of the subjects (Table 3).

## 4. Discussion

The prevalence of soil-transmitted helminthiasis in the study population was 30.3%. This classifies the community as a “moderate risk area” for preventive therapy by WHO standards [9]. Soil-transmitted helminthic infection is therefore a problem amongst primary school children aged 5–16 years in Amaruru community, in Imo State. This is similar to findings of other studies [12, 13] in other parts of Nigeria which also found helminthiasis to be common among primary school children. A similar pattern of infection is likely to occur in many other communities in the state and other parts of Nigeria. The high prevalence rate of soil-transmitted helminths in the study population could be attributable to the risk factors associated with the study population, that is, nonavailability of water supply and toilet facilities in schools. Furthermore, the majority of children belong to a lower socioeconomic class. The prevalence rate in the present study is higher than the 24.6% observed by Okolo and John [14]. The difference between this study and the Okolo study could be due to the latter study's use of a less sensitive technique (wet preparation technique) for detecting parasites [15].

The age groups of 8–10 and 11–13 years recorded higher prevalence rates of 33.9% and 31%, respectively. Children in these age groups engage in play activities in contaminated environments that could facilitate transmission of intestinal helminths. The children also tend to be less cautious of their personal hygiene because they are not old enough to understand the need for general cleanliness, unlike their counterparts in the age group of 14–16 years in which the infection rate was very low. A lower infection rate in the age group of 14–16 years may be due to the psychosocial development of the mid-adolescent, as they are more self-conscious of their personal hygiene and outward appearance to attract the opposite sex. Thus, mid-adolescents are less likely to walk around barefooted. Similar findings and reasons have been adduced by other researchers [8, 12, 13].

Pit latrines and nearby bush were the commonly used sites of sewage disposal in the study population and in agreement with the earlier report of Adefioye et al. [8]. The use of pit latrine and nearby bush reflects the poor socioeconomic status of the study subjects. Additionally, students in the schools had no access to clean water supply and toilet facility. Soil-transmitted helminths thrive and flourish in such communities.

Our study also showed that the prevalence of soil-transmitted helminthiasis was higher in males than in females. This could be because the majority of the study population were children of farmers and males usually accompany their fathers to the farm. Male children are also known to be more adventurous. This is in agreement with the report of Obiukwu et al. [12].

Multiple infections occurred in 3.5% of the subjects with confirmed soil-transmitted helminthic infection. The multiple soil-transmitted helminthic infections observed may be as a result of multiple infection risks that they are exposed to during their daily activities.

Hookworm was the most common soil-transmitted helminthic infection identified in this study and may be attributed to the rainy season when the study was conducted. The observation of increased hookworm transmission during the rainy season has been documented [16]. The plausible reason for this increased hookworm transmission during the rainy season may be that the rains disperse faeces increasing chances of parasite contact with humans.* Trichuris trichiura* infection was not observed similar to studies reported by Adefioye et al. [8].* Trichuris trichiura* generally has a very low prevalence as documented in some studies [17, 18]. However, the absence of* Trichuris trichiura* in this study may be due to the single stool examination technique utilized here. Multiple stool examinations may have improved the yield of ova of* Trichuris trichiura*.

## 5. Conclusion

The finding of a high prevalence of soil-transmitted helminthic infection amongst primary school children in Amaruru in Imo State, Nigeria, emphasises the need for potable water supply and safe sewage disposal systems in schools. There is also a need for routine regular deworming of all the students in the schools to reduce the burden of soil-transmitted helminthiasis and guarantee the delisting of the community's WHO “moderate risk area” classification status.

## Figures and Tables

**Table 1:** Sociodemographic characteristics of the study population.

Characteristic	Frequency (*n*)	Percentage (%)
Age group (years)		
5–7	34	12.0
8–10	112	39.4
11–13	121	42.6
14–16	17	6.0
Gender		
Male	151	53.2
Female	133	46.8
Socioeconomic status		
Upper class	2	0.7
Middle class	87	30.6
Lower class	195	68.7

**Table 2:** Risk factors and prevalence of soil-transmitted helminthiasis among the study subjects.

Risk factors	Helminth status			*χ* ^2^	*P* value
	Infected *n* (%)	Noninfected *n* (%)	Total *n* (%)		
Gender					
Male	58 (38.4)	93 (61.6)	151 (100)	10.09	0.001^*∗*^
Female	28 (21.1)	105 (78.9)	133 (100)		
Total	**86 (30.3)**	**196 (69.3)**	**284 (100)**		
Wearing of footwear					
Yes	66 (26.0)	188 (74.0)	254 (100)	0.15	0.700
No	20 (66.7)	10 (33.3)	30 (100)		
Total	**86 (30.3)**	**198 (69.3)**	**284 (100)**		
Hand washing after defecation					
Always	16 (27.1)	43 (72.9)	59 (100)	0.91	0.694
Occasionally	69 (31.1)	153 (69.8)	222 (100)		
Never	1 (33.3)	2 (66.7)	3 (100)		
Total	**86 (30.3)**	**198 (69.3)**	**284 (100)**		
Sewage disposal					
Water closet	1 (6.3)	15 (93.7)	16 (100)	4.79	0.09
Pit latrine	70 (32.3)	147 (67.7)	217 (100)		
Bush	15 (29.4)	36 (70.6)	51 (100)		
Total	**86 (30.3)**	**198 (69.3)**	**284 (100)**		
Age group (years)					
5–7	8 (23.5)	26 (76.5)	34 (100)	4.27	0.23
8–10	38 (33.9)	74 (66.1)	112 (100)		
11–13	38 (31.4)	83 (68.6)	121 (100)		
14–16	2 (11.8)	15 (88.2)	17 (100)		
Total	**86 (30.3)**	**198 (69.7)**	**284 (100)**		

^*∗*^Significant *P* values.

**Table 3:** Soil-transmitted helminths identified among subjects.

Species	Frequency (*n*)	Percentage (%)
Hookworm	81	94.2
*Ascaris lumbricoides *+ hookworm	3	3.5
*Ascaris lumbricoides*	2	2.3
Total	**86**	**100**
